# Fast Simulation of a Multi-Area Spiking Network Model of Macaque Cortex on an MPI-GPU Cluster

**DOI:** 10.3389/fninf.2022.883333

**Published:** 2022-07-04

**Authors:** Gianmarco Tiddia, Bruno Golosio, Jasper Albers, Johanna Senk, Francesco Simula, Jari Pronold, Viviana Fanti, Elena Pastorelli, Pier Stanislao Paolucci, Sacha J. van Albada

**Affiliations:** ^1^Department of Physics, University of Cagliari, Monserrato, Italy; ^2^Istituto Nazionale di Fisica Nucleare (INFN), Sezione di Cagliari, Monserrato, Italy; ^3^Institute of Neuroscience and Medicine (INM-6) and Institute for Advanced Simulation (IAS-6) and JARA-Institute Brain Structure-Function Relationships (INM-10), Jülich Research Centre, Jülich, Germany; ^4^RWTH Aachen University, Aachen, Germany; ^5^Istituto Nazionale di Fisica Nucleare (INFN), Sezione di Roma, Rome, Italy; ^6^Faculty of Mathematics and Natural Sciences, Institute of Zoology, University of Cologne, Cologne, Germany

**Keywords:** computational neuroscience, spiking neural networks, simulations, GPU (CUDA), primate cortex, multi-area model of cerebral cortex, message passing interface (MPI), high performance computing (HPC)

## Abstract

Spiking neural network models are increasingly establishing themselves as an effective tool for simulating the dynamics of neuronal populations and for understanding the relationship between these dynamics and brain function. Furthermore, the continuous development of parallel computing technologies and the growing availability of computational resources are leading to an era of large-scale simulations capable of describing regions of the brain of ever larger dimensions at increasing detail. Recently, the possibility to use MPI-based parallel codes on GPU-equipped clusters to run such complex simulations has emerged, opening up novel paths to further speed-ups. NEST GPU is a GPU library written in CUDA-C/C++ for large-scale simulations of spiking neural networks, which was recently extended with a novel algorithm for remote spike communication through MPI on a GPU cluster. In this work we evaluate its performance on the simulation of a multi-area model of macaque vision-related cortex, made up of about 4 million neurons and 24 billion synapses and representing 32 mm^2^ surface area of the macaque cortex. The outcome of the simulations is compared against that obtained using the well-known CPU-based spiking neural network simulator NEST on a high-performance computing cluster. The results show not only an optimal match with the NEST statistical measures of the neural activity in terms of three informative distributions, but also remarkable achievements in terms of simulation time per second of biological activity. Indeed, NEST GPU was able to simulate a second of biological time of the full-scale macaque cortex model in its metastable state 3.1× faster than NEST using 32 compute nodes equipped with an NVIDIA V100 GPU each. Using the same configuration, the ground state of the full-scale macaque cortex model was simulated 2.4× faster than NEST.

## 1. Introduction

Large-scale spiking neural networks are of growing research interest because of their ability to mimic brain dynamics and function more and more accurately. However, the task of accurately simulating natural neural networks is arduous: the human brain contains around 86 × 10^9^ neurons (Azevedo et al., 2009) and on the order of 10^4^–10^5^ synapses per neuron in the cerebral cortex (Cragg, 1975; Alonso-Nanclares et al., 2008). Moreover, the brain presents a plethora of different neurotransmitters, receptors, and neuron types connected with specific probabilities and patterns. For these reasons, even a simulation of a small fraction of the brain could be computationally prohibitive if the details of axonal and dendritic arborizations were accounted for or if, adding further complexity, accurate descriptions of biochemical processes were included. In this work, focused on enabling the simulation of multi-area cortical models on up to a few tens of compute nodes, we treat spiking simulations with point neurons and simplified synaptic rules. This level of abstraction greatly reduces computational demands while still capturing essential aspects of the neural network behavior. However, achieving short simulation times for a multi-area spiking network model is nevertheless nontrivial even on high-performance hardware with highly performant software tools.

Some simulators such as NEST (Hahne et al., 2021), NEURON (Carnevale and Hines, 2006), Brian 2 (Stimberg et al., 2019) and ANNarchy (Vitay et al., 2015) are capable of simulating a large variety of neuron and synapse models. These simulators support multithreaded parallel execution on general-purpose CPU-based systems. Furthermore, NEST and NEURON also support distributed computing *via* MPI.

Meanwhile in the last decades, to efficiently simulate large-scale neural networks in terms of both speed and energy consumption, neuromorphic hardware has been developed by taking inspiration from brain architecture. Among these systems, we can mention Loihi (Davies et al., 2018) and TrueNorth (Akopyan et al., 2015), which are entering the realm of large-scale neural network simulations, and BrainScaleS (Grübl et al., 2020), which is based on analog emulations of simplified models of spiking neurons and synapses, with digital connectivity. The system enables energy-efficient neuronal network simulations, offering highly accelerated operations. Another promising project in this field is SpiNNaker (Furber et al., 2014), which recently achieved biological real-time simulations of a cortical microcircuit model (Rhodes et al., 2020) proposed by Potjans and Diesmann (2014) (which has since been simulated sub-realtime with NEST (Kurth et al., 2022) and with an FPGA-based neural supercomputer (Heittmann et al., 2022). This result was made possible by its architecture designed for efficient spike communication, performed with an optimized transmission system of small data packets. BrainScaleS and SpiNNaker are freely available to the scientific community through the EBRAINS Neuromorphic Computing service. Nevertheless, neuromorphic systems still require a significant amount of system-specific skills. Even if the simulation speed they can provide is impressive, the flexibility and simplicity of programming environments available for such neuromorphic systems are still low compared to their general-purpose counterparts. On neuromorphic systems adopting analog design techniques, advantages in speed, area, and energy consumption are associated with the difficulties of managing manufacturing fluctuations, unavoidable in analog substrates, and with the effects of electronic noise emerging in the dynamics of analog circuits. Porting neural simulations from digital systems to analog neuromorphic platforms is not a trivial task. Overcoming such difficulties and turning them into advantages is an emerging field of research (Wunderlich et al., 2019). Furthermore, as soon as the number of synapses established by each neuron reaches biological scales (i.e., several thousands per neuron), the current generation of neuromorphic systems often experience significant slowdown, whereas a new generation capable of coping with such issues is still under development. For example, in its maximum configuration, the first-generation BrainScaleS system hosts 1 billion synapses and 4 million neurons (250 synapses/neuron) on 20 silicon wafers (Güttler, 2017), and a similar synapse-per-neuron ratio is the sweet spot for optimal execution on SpiNNaker, well below the typical 10K synapses/neuron characteristic for pyramidal cortical neurons or >100K synapses/neuron sported by cerebellar Purkinje cells.

Lately some systems based on graphical processing units (GPUs) have emerged (Sanders and Kandrot, 2010; Garrido et al., 2011; Brette and Goodman, 2012; Vitay et al., 2015; Yavuz et al., 2016). These systems grant a higher flexibility compared to neuromorphic systems, because of the current technological constraints of the latter and because of the software support offered by platforms like CUDA (Compute Unified Device Architecture) (Sanders and Kandrot, 2010), created by NVIDIA to take advantage of the large compute resources of GPUs. As a matter of fact, spiking neural network simulations could reap large benefits from the high degree of parallelism of GPU systems, which allows for thousands of simultaneous arithmetic operations even for a single GPU. However, the effective speed-up made possible by parallelization on GPUs can be limited by sequential parts and operations like I/O of spike recordings and feeding inputs into the network model, which inevitably require data transfer between CPU and GPU memory.

Among GPU-based simulators we can mention CARLSim4 (Chou et al., 2018), a spiking neural network simulator written in C++ with a multi-GPU implementation, and NCS6 (Hoang et al., 2013), a CPU/GPU simulator specifically designed to run on high-performance computing clusters. More recently, CoreNEURON (Kumbhar et al., 2019) was developed as an optimized compute engine for the NEURON simulator. It is able to both reduce memory usage and increase simulator performance with respect to the NEURON simulator by taking advantage of architectures like NVIDIA GPUs and many-core CPUs. One of the most popular GPU-based simulators for spiking neural networks is GeNN (Yavuz et al., 2016), which has achieved fast simulations of the cortical microcircuit model of Potjans and Diesmann (Knight and Nowotny, 2018; Knight et al., 2021). Recently the same simulator, running on a single high-end GPU, has shown better performance compared to what was obtained with a CPU-based cluster (Knight and Nowotny, 2021) in the simulation of a multi-area spiking network model of macaque cortex (Schuecker et al., 2017; Schmidt et al., 2018a,b). This result was reached thanks to the procedural connectivity approach, consisting in generating the model connectivity and its synaptic weights only when spikes need to be transmitted, without storing any connectivity data in the GPU memory. As a matter of fact, one of the most constraining features of GPUs is the size of the built-in memory, which in spiking neural network simulations can be a severe limitation. The possibility of generating the connections on demand enables performing a large-scale simulation even with a single GPU. However, procedural connectivity is a suitable approach only with static synapses. Indeed, plastic synapses require data to be stored since their synaptic weights change their value during the simulation. The inclusion of plastic synapses is essential for many investigations, e.g., when learning or the interplay between synaptic changes and brain dynamics are of interest (Capone et al., 2019; Golosio et al., 2021a). GeNN allows and supports models with synaptic plasticity, but for such models the procedural connectivity approach is thus prevented.

NEST GPU (previously named NeuronGPU) (Golosio et al., 2021b) is a GPU-MPI library written in CUDA for large-scale simulations of spiking neural networks, which was recently included in the NEST Initiative with the aim of integrating it within the NEST spiking network simulator, in order to allow for simulations on GPU hardware. In this work we evaluate the performance of NEST GPU on simulations that exploit multiple GPUs on MPI clusters. The library implements a novel MPI-optimized algorithm for spike communication across processes that also leverages some of the delivery techniques already investigated for CPU-based distributed computing platforms. Currently, NEST GPU exploits the neuron distribution among processes as described in Pastorelli et al. (2019): neurons are allocated on processes taking into account their spatial locality, instead of using a round-robin approach. Spike delivery takes advantage of this distribution mode, resulting in an efficient and optimized algorithm. NEST GPU supports a large variety of neuron models and synapses, both static and plastic. In this work we compare the outcomes of NEST GPU and NEST for the full-scale multi-area spiking network model of macaque cortex simulated on a high-performance computing (HPC) cluster with both GPU- and CPU-equipped compute nodes. To this end the distributions of firing rates, coefficients of variation of interspike intervals (CV ISI), and Pearson correlations between spike trains obtained by the two simulators are examined. We further evaluate the performance in terms of simulation time per second of biological activity.

## 2. Materials and Methods

### 2.1. NEST GPU Spike Communication and Delivery Algorithm

In this section the algorithm exploited by NEST GPU for spike communication between MPI processes and for spike delivery is briefly introduced. For an in-depth description of the spike delivery algorithm please see Golosio et al. (2021b).

In NEST GPU, the output connections of each neuron (or other spiking device) are organized in groups, all connections in the same group having the same delay. For each neuron there is a spike buffer, which is structured as a queue used to store the spikes emitted by the neuron. Each spike is represented by a structure with three member variables: a time index *t*_*s*_, which starts from 0 and is incremented at every time step; a connection group index *i*_*g*_, which also starts from zero and is increased every time the spike matches a connection group, i.e., when the time index corresponds to the connection group delay; and a multiplicity, i.e., the number of spikes emitted by the neuron in a single time step. Keeping a connection group index and having connection groups ordered according to their delays is useful for reducing the computational cost, because it avoids the need for a nested loop to compare the time index of the spike with all the connection delays. When the time index of a spike matches a connection group delay, spike information (i.e., source neuron index, connection group index, multiplicity) is inserted in a global spike array and the connection group index is increased. A spike is removed from the queue when *i*_*g*_ becomes greater than the number of connection groups of that neuron, i.e., when the time index becomes greater than the maximum delay. The final delivery from the global spike array to the target neurons is done in a loop, so no additional memory is required. When a source neuron is connected to target neurons belonging to a different MPI process, a spike buffer, similar to the local one, is created in the remote MPI process. When the source node fires a spike, this is sent to the spike buffer of the remote MPI process, which will deliver the spike to all target neurons after proper delays. The remote spikes, i.e., the spikes that must be transferred to remote MPI processes, are communicated through non-blocking MPI send and receive functions at the end of every simulation time step. Let *N* be the number of MPI processes. The whole procedure consists of three stages:

1. Each MPI process initiates a non-blocking receive (MPI_Irecv) on *N*−1 receiving buffers (one for each remote MPI process), so that all receiving buffers are ready more or less simultaneously;
2. Each MPI process initiates forwarding of the remote spikes to all other *N*−1 processes by calling a non-blocking send (MPI_Isend);
3. Each MPI process initializes a list with the indexes of the other *N*−1 processes, and starts checking all the items in the list in an endless loop with MPI_Test. When the transfer from the *i*-th MPI process is complete, the corresponding index *i* is removed from the list. The loop is interrupted when the list is empty.

**Figure 1:**
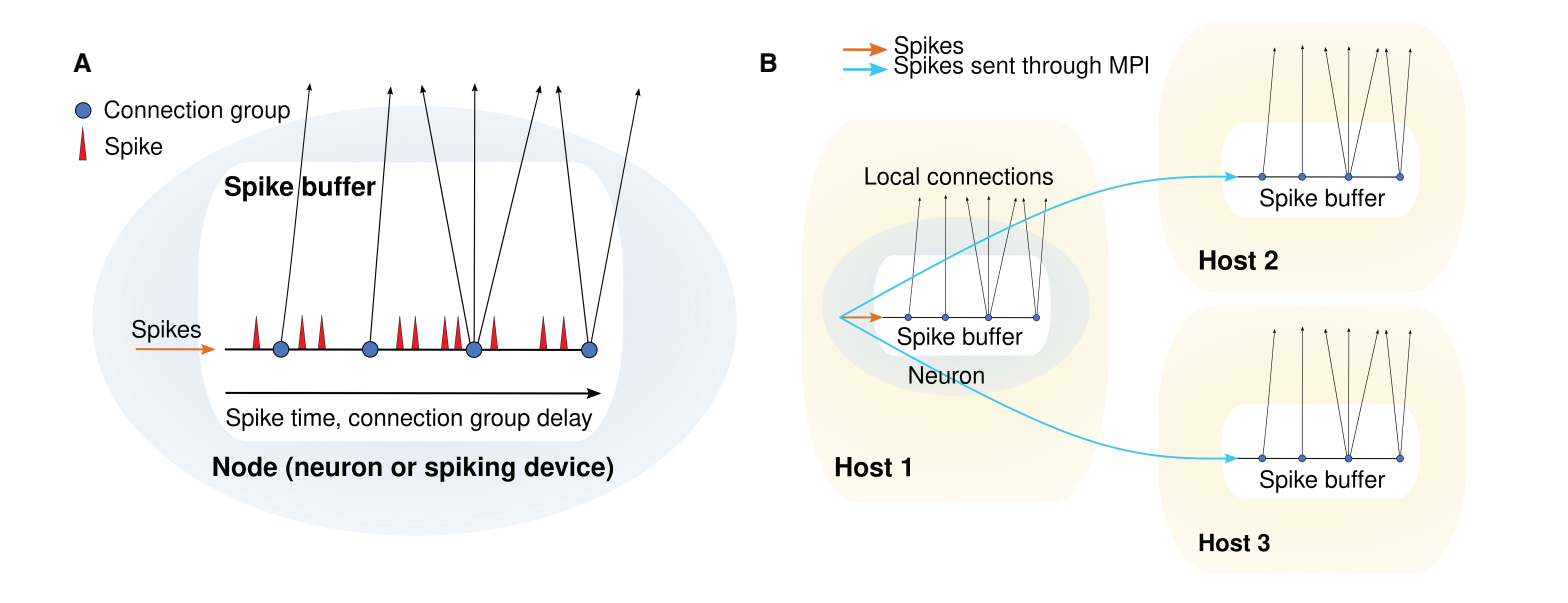
Spike handling and delivery schemes. **(A)** Structure of a single spike buffer. **(B)** Schematic depicting MPI communication between spike buffers for different hosts.

The spike buffer for a single network node and the spike handling and delivery for multiple MPI processes are depicted in Figure 1.

### 2.2. NEST GPU Spike Recording Algorithm

In this section the NEST GPU algorithm for spike time recording is introduced. The spike times are initially recorded in the GPU memory in a two-dimensional array, with the number of rows equal to the number of neurons and the number of columns equal to the maximum number of spikes that can be recorded before each extraction. Since the number of spikes per neuron is typically much smaller than the maximum, most entries of this array are zero. To compress the information, the spike times are periodically packed into contiguous positions of a one-dimensional buffer, which is copied from GPU memory to RAM along with a one-dimensional array indicating the positions at which the spikes for each neuron start. The packing algorithm works as follows:

1. Let *N*_*i*_ be the number of recorded spikes of the *i*-th neuron, and *C*_*i*_ its cumulative sum (also called prefix scan):

(1) $$\begin{array}{r} C_{0}=0;\;C_{i}=\overset{i-1}{\underset{k=0}{\sum }}N_{k}\;\text{for}i=1,...,n \end{array}$$

where *n* is the number of neurons. Note that *C*_*i*_ has *n*+1 elements, one more than *N*_*i*_, and that it is sorted by construction. *C*_*i*_ is computed in parallel with CUDA using the algorithm described by Nguyen (2007, Chapter 39) as implemented in https://github.com/mattdean1/cuda. The last element of *C*_*i*_, *N*_tot_ = *C*_*n*_, is the total number of recorded spikes of all neurons;
2. Let *t*_*i, j*_ be the time of the *j*-th recorded spike of the *i*-th neuron. The packed spike array *A*_*m*_ (*m* = 0, …, *N*_tot_−1) is computed from *t*_*i, j*_ using a one-dimensional CUDA kernel with *N*_tot_ threads. *m* is set equal to the thread index. Since *C*_*i*_ is sorted, a binary-search algorithm can be used to find the largest index *i* such that

(2) $$\begin{array}{r} C_{i}\leq m<C_{i+1} \end{array}$$

*C*_*i*_ will be the index of the first spike of the *i*-th neuron in the packed spike array, therefore the spike *m* in this array will correspond to the spike *i, j* in the original two-dimensional array *t*_*i, j*_, where *j* is simply

(3) $$\begin{array}{r} j=m-C_{i} \end{array}$$

Once *i* and *j* are computed from *m*, it is possible to set

(4) $$\begin{array}{r} A_{m}=t_{i,j} \end{array}$$

Packing of recorded spikes and transfer to the RAM can be performed after a certain number of simulation time steps depending on GPU memory availability.

### 2.3. Multi-Area Model

**Figure 2:**
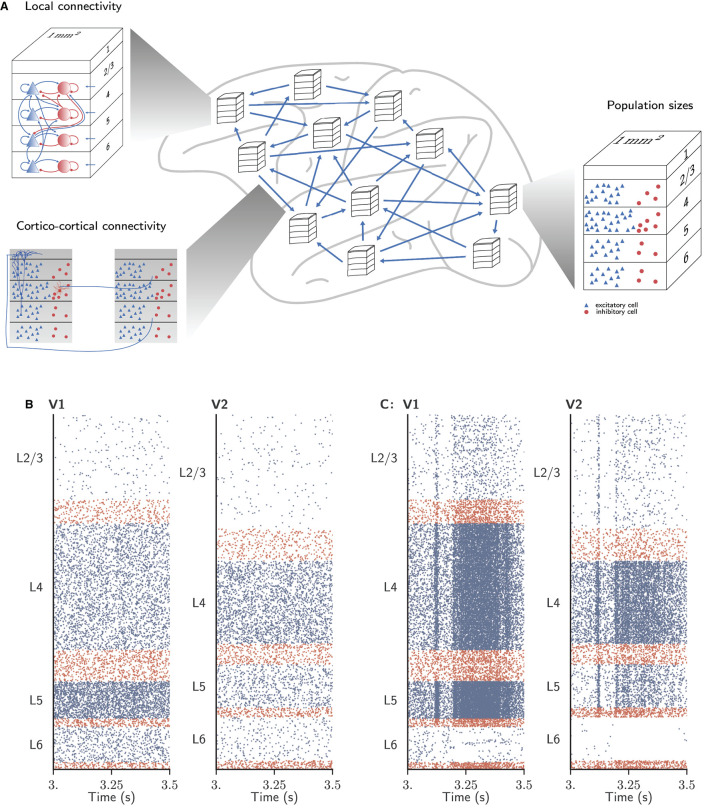
Spiking neuronal network model used to evaluate simulator performance in this study. **(A)** Schematic overview of the model. The multi-area model represents 32 areas of macaque vision-related cortex, each modeled by four cortical layers with a size of 1mm^2^. Local connectivity, cortico-cortical connectivity, and population sizes are adapted for each area. **(B)** Network activity of areas V1 and V2 in the ground state. **(C)** Network activity of the same areas in the metastable state. Figure adapted from Schmidt et al. (2018a) and Schmidt et al. (2018b).

We consider the dynamics of a model of all vision-related areas in one hemisphere of macaque cortex (Schmidt et al., 2018a,b) (Figure 2). Here, we briefly summarize the model; all details and parameter values can be found in the original publications. Following the parcellation of Felleman and Van Essen (1991), the model includes 32 areas that either have visual function or are strongly interconnected with visual areas. To yield a tractable model size, only 1 mm^2^ of cortex is represented within each area, albeit with the full local density of neurons and synapses. This leads to a total of about 4.1 million neurons and 24 billion synapses. The areas have a laminar structure, layers 2/3, 4, 5, and 6 each containing one population of excitatory (E) and one population of inhibitory (I) neurons (area TH lacks layer 4); hence the total number of populations in the network is 254. The neuron model is the leaky integrate-and-fire model with exponential current-based synapses, and all neurons have the same electrophysiological parameter values. The initial membrane potentials are normally distributed. Input from non-modeled brain regions is represented by homogeneous Poisson spike trains with area-, layer- and population-specific rates.

The numbers of neurons are determined from a combination of empirically measured neuron densities, cytoarchitectural type definitions of areas, and the thicknesses of the cortical layers. The connectivity of the local microcircuits consists of scaled versions of the connectivity of a microcircuit model of early sensory cortex (Potjans and Diesmann, 2014). The inter-area connectivity is based on axonal tracing data collected in the CoCoMac database (Bakker et al., 2012), complemented with the quantitative tracing data of Markov et al. (2011, 2014). Gaps in the data are filled by predictions of overall connection densities from inter-area distances, and laminar patterns from relative neuron densities of source and target areas. The synapses are statistically mapped to target neurons based on the extent of the dendritic trees of morphologically reconstructed neurons of each type in each layer (Binzegger et al., 2004). A mean-field-based method slightly adjusts the connectivity to support plausible spike rates (Schuecker et al., 2017).

When the cortico-cortical synapses have the same strength as the local synapses, this leads to a stationary “ground state” of activity without substantial rate fluctuations or inter-area interactions (Figure 2B). As this state does not match experimental resting-state recordings of spiking activity and functional connectivity between areas, the cortico-cortical synaptic strengths are increased, especially onto inhibitory neurons, in order to generate substantial inter-area interactions while maintaining balance. Poised just below a transition to a high-activity state, the spiking activity is irregular with low synchrony apart from population events of variable duration. In this “metastable state” (Figure 2C), aspects of both microscopic and macroscopic resting-state activity in lightly anesthetized monkeys are well reproduced: the spectrum and spike rate distribution of the modeled spiking activity of primary visual cortex (V1) are close to those from parallel spike train recordings (Chu et al., 2014a,b); and the functional connectivity between areas approximates that obtained from fMRI recordings (Babapoor-Farrokhran et al., 2013).

For further details we refer to the original publications (Schmidt et al., 2018a,b).

## 3. Results

In this section we first verify the correctness of the simulations performed by NEST GPU, using NEST 3.0 as a reference. Afterwards, the performance evaluation is presented in terms of build (i.e., network construction) and simulation time.

To this end, we used the HPC cluster JUSUF (von St. Vieth, 2021). In particular, the NEST GPU simulations employed 32 accelerated compute nodes, each of them equipped with two AMD EPYC 7742 (2 × 64 cores, 2.25 GHz), 256 GB of DDR4 RAM (3,200 MHz), and an NVIDIA V100 GPU with 16 GB HBM2e; inter-node communication is enabled *via* InfiniBand HDR100 (Connect-X6). The NEST simulations were run on standard compute nodes of the HPC cluster JURECA-DC (Thörnig and von St. Vieth, 2021), which uses the same CPUs and interconnect as JUSUF but has 512 GB of DDR4 RAM per node available.

### 3.1. Comparison of Model Results Between NEST and NEST GPU

In Golosio et al. (2021b) some of us have compared the simulation outcomes between NEST GPU and NEST for the cortical microcircuit model of Potjans and Diesmann (2014), showing an optimal match between the results of both simulators. The validation approach follows that of van Albada et al. (2018) and Knight and Nowotny (2018). In this section we present a similar procedure in order to validate the NEST GPU outcome for the multi-area model considered here.

Firstly, for each of the executed simulations, we simulated 10 s of biological activity of the full-scale multi-area model in both NEST and NEST GPU. All the simulations were performed with a time step of 0.1 ms. We simulated both the ground state (showing asynchronous irregular spiking with stationary rate) and the metastable state of the model (better representing the resting-state activity of the cortex) in order to compare the results of both configurations. To avoid transients due for instance to initial synchronization, a pre-simulation time of 500 ms was employed for all the simulations. This enhances the independence of the derived activity statistics from the total simulation time.

We executed 10 simulations for each simulator, recording the spike times. The 10 simulations differ in the chosen seed for the random number generation, so that there is no pairwise matching of seeds between NEST and NEST GPU simulations. Furthermore, we performed another set of 10 simulations with NEST to estimate the differences that arise only because of the different seeds used. Taking their outcome as a reference for both NEST GPU and NEST simulations, it was possible to evaluate NEST-NEST and NEST-NEST GPU comparisons.

To compare the simulation outcomes using the recorded spike times, we selected and extracted the distributions of three quantities for each population:

- The time-averaged firing rate of each neuron;
- The coefficient of variation of inter-spike intervals (CV ISI), i.e., the ratio between the standard deviation and the average of inter-spike time intervals of each neuron;
- The pairwise Pearson correlation between the spike trains obtained from a subset of 200 neurons for each population, in order to grant a reasonable computing time.

The spike trains were binned with a time step of 2 ms, corresponding to the refractory time, so that at most one spike could occur in each bin. Considering a binned spike train *b*_*i*_ for neuron *i* with mean value μ_*i*_, the correlation coefficient between two spike trains *b*_*i*_ and *b*_*j*_ is defined as:


(5) $$\begin{array}{r} C\left[i,j\right]=\langle b_{i}-\mu_{i},b_{j}-\mu_{j}\rangle /\sqrt{\langle b_{i}-\mu_{i},b_{i}-\mu_{i}\rangle \cdot \langle b_{j}-\mu_{j},b_{j}-\mu_{j}\rangle } \end{array}$$

where 〈, 〉 represents the scalar product. Hence a 200 × 200 matrix is built and the distribution of the Pearson correlations can be evaluated as the distribution of the off-diagonal elements. All aforementioned distributions were computed using the Elephant package (Denker et al., 2018).

The raw distributions were smoothed using Kernel Density Estimation (KDE) (Rosenblatt, 1956; Parzen, 1962). The KDE method was applied with the sklearn.neighbors.KernelDensity function of the scikit-learn Python library (Pedregosa et al., 2011) (version 0.24.2). Specifically, we performed KDE with a Gaussian kernel, optimized with a bandwidth obtained using the Silverman method (Silverman, 1986).

**Figure 3:**
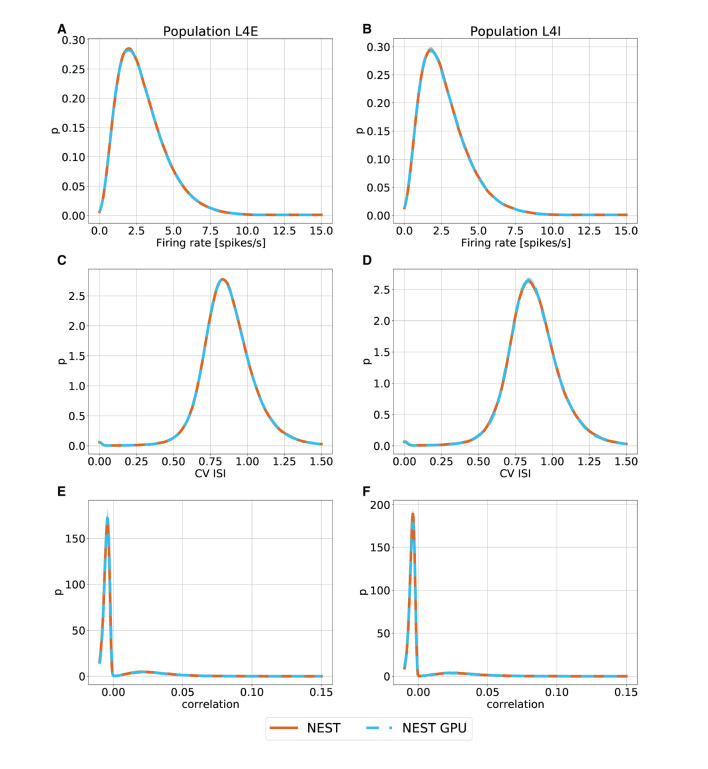
Ground state distributions of firing rate **(A,B)**, CV ISI **(C,D)** and Pearson correlation of the spike trains **(E,F)** for the populations L4E and L4I of area V1. The distributions are averaged over 10 simulations with NEST (orange) and NEST GPU (sky blue). Every averaged distribution has an error band representing its standard deviation.

**Figure 4:**
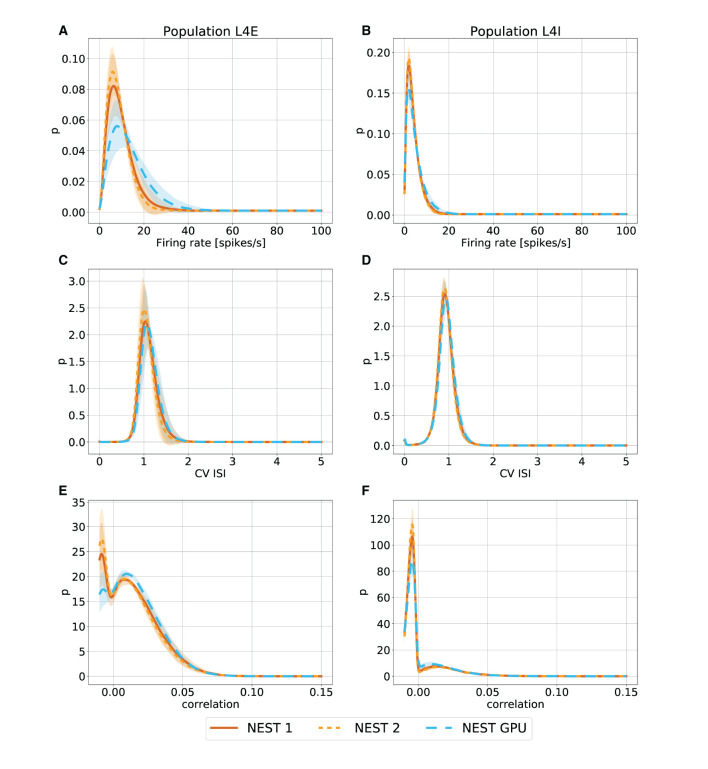
Metastable state distributions of firing rate **(A,B)**, CV ISI **(C,D)** and Pearson correlation of the spike trains **(E,F)** for the populations L4E and L4I of area V1. The distributions are averaged over 10 simulations with NEST (orange lines) and NEST GPU (sky blue dashed line). Every averaged distribution has an error band representing its standard deviation. An additional set of NEST simulation distributions is also shown.

With this procedure we obtained 762 distributions for each simulation. For each of the 254 populations we determined the average and standard deviation of these distributions across each set of 10 simulations. To gain an impression of the similarity of the simulation outcomes of NEST and NEST GPU, example distributions are shown in Figures 3, 4.

As can be observed, the distributions obtained with the two simulators closely match each other in the ground state (Figure 3), and also the error bands are negligible because of the small variability of the state. In the metastable state, the variability between the NEST and NEST GPU distributions is larger (Figure 4). Due to the increased variability, we decided to depict an additional NEST distribution to show the substantial fluctuations that can arise between two sets of NEST simulations.

**Figure 5:**
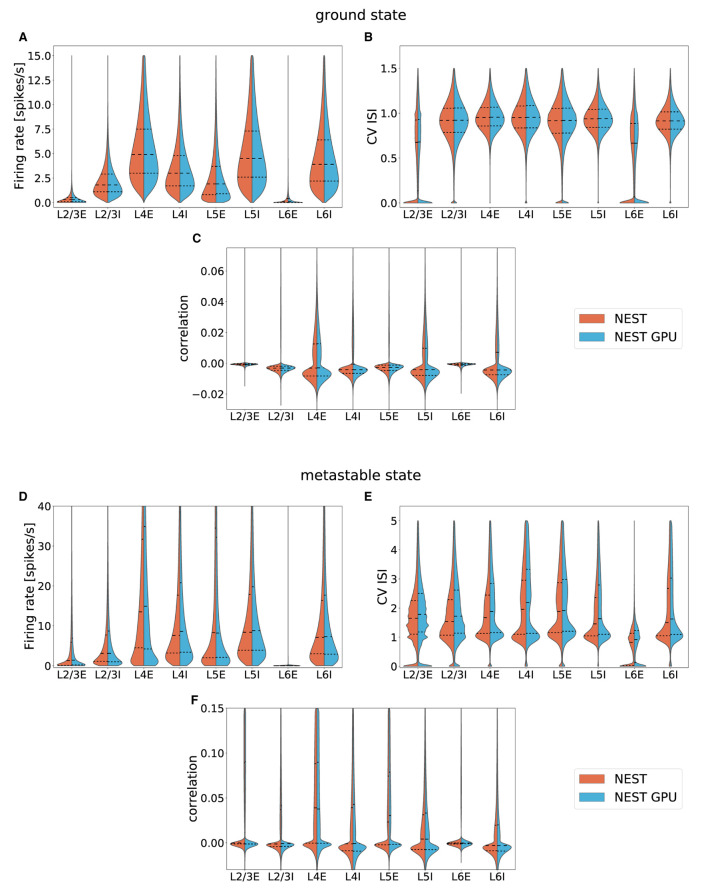
Averaged distributions of the ground state and the metastable state of the model for all 32 areas obtained using NEST (orange, left side) and NEST GPU (sky blue, right side) and compared with split violin plots. The central dashed line represents the distribution's median, whereas the other two dashed lines represent the interquartile range. **(A,D)** average firing rate, **(B,E)** average CV ISI, **(C,F)** average Pearson correlation of the spike trains.

To provide an overview over the distributions for the entire model, averaged distributions for each layer and area were computed. These data were plotted with the seaborn.violinplot function of the Seaborn Python library (Waskom, 2021) (version 0.11.1), which returns KDE-smoothed distributions optimized with the Silverman method, matching our calculation of the distributions. The distributions thus obtained were compared by placing them side by side in the split violin plots shown in Figure 5, also showing median and interquartile range for every distribution.

The area-averaged distributions compared in Figure 5 are nearly indistinguishable. The same holds for each of the 254 population-level distributions separately^1^.

To quantify the similarity between the distributions, the Earth Mover's Distance (EMD) was computed. This metric evaluates the distance between two probability distributions, and its name stems from an analogy with the reshaping of soil. The two distributions may be thought of as, respectively, a given amount of earth located in a certain space and the same amount of earth that has to be arranged properly. The Earth Mover's Distance can thus be seen as the minimum amount of work needed to obtain the desired distribution from the original one. It is equivalent to the 1st Wasserstein distance between two distributions (see Supplementary Material). In this work it has been computed using the scipy.stats.wasserstein_distance function of the Python scientific library SciPy (Virtanen et al., 2020) (version 1.5.2). We opted for this measure instead of the Kullback-Leibler divergence adopted in the procedure described in Golosio et al. (2021b) because of the metric properties of the EMD, which makes it not only more specific in detecting the degree of dissimilarity among distributions but also symmetric.

To verify the equivalence between the simulators we analyzed the box plots obtained from the set of 10 EMD values for each population, given by the pairwise comparison of each of the 10 simulations. This way, we take into consideration the possible variability due to the different random number generator seeds. The random connectivity, membrane potential initialization, and external drive mean that one expects a nonzero EMD between simulations with different random seeds even with the same simulator. Furthermore, the different order of the operations in the two simulators combined with the chaotic dynamical state imply that nonzero differences would be expected even with the same random seeds for different simulators. Since EMD has the same units as the variables over which the distributions are computed, it is possible to directly estimate the relevance of the corresponding values.

**Figure 6:**
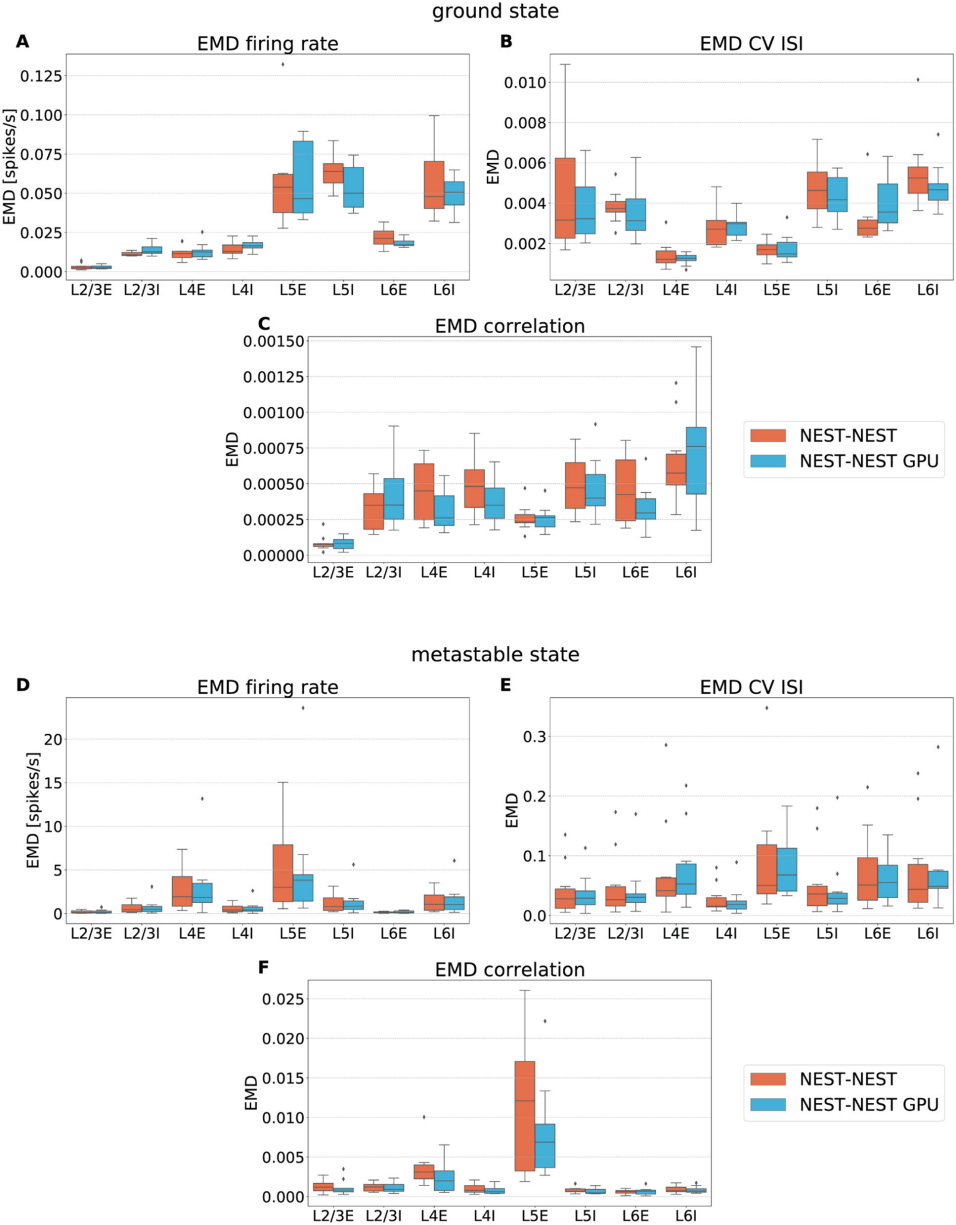
Earth Mover's Distance between distributions of firing rate **(A,D)**, CV ISI **(B,E)** and correlation of the spike trains **(C,F)** obtained for area V1 of the model in the ground state and the metastable state. NEST-NEST (orange, left) and NEST-NEST GPU (sky blue, right) data are placed side by side.

Figure 6 shows the EMD box plots obtained from the comparisons NEST-NEST and NEST-NEST GPU for the three distributions calculated for area V1, respectively, for the ground state and the metastable state.

The EMD values for the NEST-NEST GPU comparison are distributed similarly to those for the NEST-NEST comparison, meaning that the differences that arise due to the choice of simulator are statistically similar to those between NEST simulations with different random number generator seeds. Thus, using NEST GPU instead of NEST (with different random numbers) does not add variability compared to using different random seeds with the same simulator. This is a further indication that NEST and NEST GPU yield statistically closely similar results. EMD values obtained by the comparison of the ground state distributions are smaller than the EMD values obtained for the metastable state. This is due to the increased fluctuations in the latter state of the model. In some cases, the whiskers for the NEST-NEST and NEST-NEST GPU comparisons have different extents. This may be related to long-tailed distributions of the corresponding activity statistics, especially for correlations (cf. Figure 5). Differences in the tails of the distributions caused by only a few data points can lead to large differences in EMD values because the probability mass needs to be moved over large distances to turn one distribution into another. However, the EMD values are marginal compared to the values within the distributions shown in Figure 5, revealing a negligible difference between the NEST and NEST GPU simulation results. This statement is also true for the other areas of the model, as shown in the Supplementary Material.

### 3.2. Performance Evaluation

Hitherto we showed that NEST and NEST GPU simulation outcomes are comparable. In this section the performance of NEST GPU is evaluated and compared with that of NEST 3.0.

We divided the total execution time into build and simulation time. The former includes the time needed to allocate memory for the network components (i.e., neurons, synapses, and all devices, such as Poisson generators and spike detectors), and to establish the connections. The simulation time measures how long it takes to propagate the network dynamics for the specified amount of biological time once the model has been set up.

The simulation time for NEST and NEST GPU was further divided to reflect the following subtasks:

- Delivery, describing the time for local spike handling and delivery;
- MPI communication, describing the time for remote spike handling and delivery;
- Collocation, i.e., the time employed for the preparation of the MPI send buffers;
- Update, i.e., the dynamics update time;
- Other, a general subtask in which other contributions to the overall simulation time are taken into account.

As reported in Golosio et al. (2021b), NEST GPU creates the model connections in the RAM, and thereafter copies them to the GPU memory. For this reason, the build phase, i.e., the phase related to the network construction, does not take advantage of any speed-up due to the use of GPUs. However, the build phase does not depend on the biological time, meaning that the more biological time is simulated, the less relevance the build time has for the overall duration of the simulation.

The simulations performed on JUSUF by NEST GPU used 32 compute nodes with one MPI process each and 8 threads per MPI process. It should be noted that while NEST uses MPI and thread parallelism during both build and state propagation phases, the number of threads per MPI process in NEST GPU affects only the build time, because the connections are initially created in parallel by different OpenMP threads in CPU memory, as stated above. This parallel setup, which permits the simulation of an area for each compute node, was the most efficient in terms of compute time, because the NVIDIA V100 GPU memory can hold one model area at most and also because in this setup only inter-area communications have to be carried out by MPI. Indeed, it is known that one of the most significant bottlenecks in parallel computation is the communication between MPI processes (Marjanović et al., 2010), and herein the way NEST GPU handles spike delivery and distributes model areas between MPI processes (i.e., an area for each MPI process) grants an efficient parallel optimization.

Performance was evaluated using 10 simulations of 10 s of biological time for both NEST and NEST GPU, averaging over random number generator seeds. In contrast to the previous simulations, spike recording was disabled. To obtain a single set of values for each simulation we performed time measurement on each employed compute node separately and then we averaged the obtained values. Since the MPI processes are synchronized by NEST GPU after each simulation time step, the overall simulation time for each node is the same; however, the time taken by individual subtasks differs somewhat across the MPI processes due to the differences between the areas of the model, such as number of neurons, density of connections, and activity rate. These subtask differences across the MPI processes are discussed later in this section. Once we extracted a single set of timings for each simulation we computed their mean and standard deviation to obtain a unique set of values.

**Figure 7:**
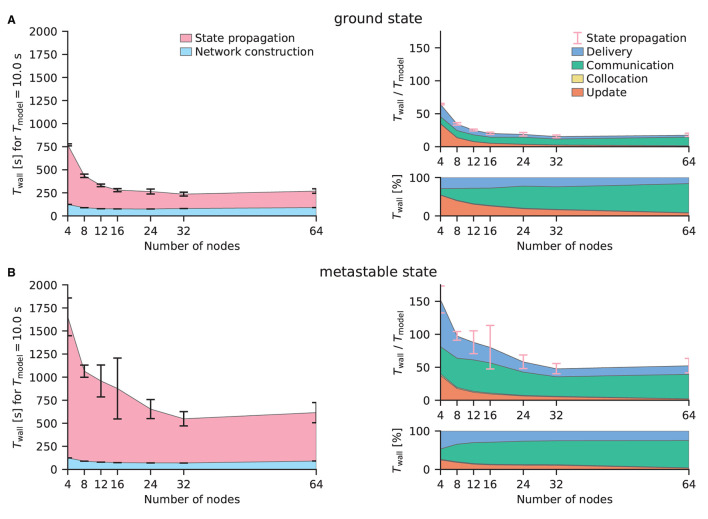
Strong-scaling performance of the multi-area model in its ground and metastable states on JURECA-DC using NEST 3.0. **(A)** Simulated with parameters inducing stable ground state activity in the network. The left sub-panel displays the absolute wall-clock time *T*_wall_ for the network construction and state propagation in ms for a biological model time *T*_model_ = 10s. Error bars indicate the standard deviation of the performance across 10 repeat simulations with different random seeds, the central points of which show the respective mean values. Error bars are shown in pink in the right panels to indicate that they are for the state propagation phase as a whole; the corresponding standard deviations are the same as in the left panels. The top right sub-panel presents the real-time factor defined as *T*_wall_/*T*_model_. Detailed timers show the absolute (top right) and relative (bottom right) time spent in the four different phases of the state propagation: update, collocation, communication, and delivery. Where the collocation phase is not discernible, this is due to its shortness. **(B)** Simulated with parameters inducing a metastable state with population bursts of variable duration. Same arrangement as **(A)**.

Figure 7 shows the performance benchmarks of the multi-area model on CPUs conducted on JURECA-DC using the benchmarking framework beNNch (Albers et al., 2022). For both network states, the optimal configuration of the hybrid parallelization is achieved with 8 MPI processes per node and 16 threads per task, thus making use of every physical core of the machine while avoiding hyperthreading. NEST distributes neurons in a round-robin fashion across virtual processes. This implements a simple form of static load balancing as neuronal populations are distributed evenly. Larger error bars in Figure 7B demonstrate the increased dependence on initial conditions and decreased stability of network activity of the metastable state. For the network simulations of the ground and metastable states, the scalings plateau at 12 nodes and 32 nodes, respectively. As discussed in Jordan et al. (2018), plateau is expected in strong scaling experiments once the MPI communication dominates. Figure 7 shows that indeed all contributions except the communication get smaller for increasing numbers of MPI processes.

In the ground state simulations, comparing the configurations with 32 nodes, the network construction times were 951 ± 29 s and 80 ± 7 s (mean ± st.dev.) for NEST GPU and NEST, respectively. Simulations of the multi-area model in its metastable state revealed similar network construction times of 957±41 s for NEST GPU and 69.5±0.4 s for NEST.

In terms of state propagation time, ground state simulations took 6.5 ± 0.1 s using NEST GPU, whereas NEST took 15.6 ± 2.1 s, both measured per second of biological model time. In the metastable state NEST GPU was able to compute a second of biological activity in 15.3 ± 0.9 s, whereas NEST took 47.9 ± 7.7 s. The longer simulation time taken for the metastable state is explained by the higher firing rates and synchrony in this state.

In case of enabled spike recording using NEST GPU the simulation time increases up to 5% when recording from all neurons. In these simulations, packing of recorded spikes and transfer to the CPU memory is performed every 2,000 simulation time steps (i.e., every 200 ms of biological time). This overhead is strongly dependent on the model simulated and the amount of GPU memory available. In fact a larger GPU memory would support larger buffers of recorded spikes, diminishing the frequency of copy operations from GPU memory to CPU memory. Furthermore, the overhead can be reduced by recording spikes from only a fraction of the neurons.

**Figure 8:**
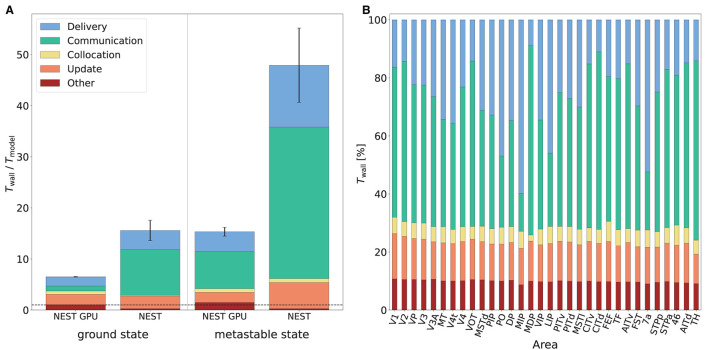
Contributions to the simulation time of the multi-area model. **(A)** Contributions to the simulation time in the ground state and the metastable state for NEST and NEST GPU measured with the real-time factor. Error bars show the standard deviation of the overall performance across 10 simulations with different random seeds. The plot shows the performance obtained by NEST GPU and NEST in the 32-node configuration. NEST simulations were performed on JURECA-DC using 8 MPI processes per node and 16 threads per task, whereas NEST GPU simulations were performed on JUSUF using one MPI process per node and 8 threads per task. The black dashed line indicates the biological time. **(B)** Relative contributions to the simulation time of the multi-area model in the metastable state for every area (i.e., for every MPI process) in a NEST GPU simulation.

Figure 8A shows the various contributions to the simulation time for NEST and NEST GPU. The main difference between the simulators appears in the time taken by spike communication, evincing the advantage of exploiting a neuron distribution among MPI processes that takes into account spatial locality. The round-robin distribution of neurons in NEST necessitates a larger degree of parallelization and hence communication to reach optimal performance. This increased communication is needed regardless of whether MPI or OpenMP parallelism is used. Indeed, replacing the 8 MPI processes per node by a further 8 threads incurs an even greater performance penalty (data not shown). We here compare both simulators in configurations which yield optimal performance.

The relative contributions of the various phases do not differ strongly between the ground and metastable states. The contribution of the communication of spikes between different MPI processes for the metastable state of the model is around 8.0 and 29.7 s per second of biological time for NEST GPU and NEST, respectively. The contribution of update, delivery, and other operations, excluding the communication of spikes between different MPI processes, is around 7.3 s for NEST GPU and 18.0 s for NEST. We can therefore observe that the better performance of NEST GPU compared to NEST is mainly due to a reduction in the communication time of the spikes between MPI processes, although there is an improvement also in the time associated with the update and delivery of local spikes.

Regarding the differences in computation time across MPI processes in NEST GPU, as mentioned above, the time taken by individual subtasks can vary across MPI processes because of differences between the areas of the model. However, since MPI processes are synchronized at the end of every simulation time step, the overall simulation time shown by every MPI process is the same. The resulting latency due to the difference between model areas is embedded in the Communication subtask. We measured that, within a simulation, the contribution of the spike communication between the 32 MPI processes (i.e., the 32 areas of the model) can vary up to 25% with respect to its average shown in Figure 8A and the contribution of the local spike delivery subtask shows comparable variations. The rest of the subtasks (i.e., Collocation, Update and Other) do not change significantly across the MPI processes, as shown in Figure 8B.

## 4. Discussion

In this work we have compared the simulators NEST GPU and NEST on a full-scale multi-area spiking network model of macaque cortex with 4.1 million neurons and 24 billion synapses (Schmidt et al., 2018a,b). As described at the beginning of the Results section, the NEST GPU simulations used 32 nodes of the HPC cluster JUSUF, each node of which is equipped with an NVIDIA V100 GPU. The NEST simulations used 32 nodes of the JURECA-DC cluster, each of which is equipped with two AMD EPYC 7742 CPUs. We have considered both the ground state of the model and the metastable state, where the latter better represents *in vivo* cortical activity thanks to stronger inter-area connections.

Figure 5, showing the averaged distributions of firing rate, CV ISI, and Pearson correlation obtained with NEST and NEST GPU, exhibits the compatibility between the outcomes of the two simulators in both states of the network. We have also quantified the differences that arise between a NEST and a NEST GPU simulation using the Earth Mover's Distance (EMD) metric. Specifically, we used EMD to evaluate the differences between the distributions obtained for each population with the two simulators. The results of this analysis show that the differences between NEST and NEST GPU simulations are comparable to those between multiple NEST simulations differing only in terms of their random seeds.

Regarding simulation performance, we observed that the build time of the multi-area model simulations is substantially higher using NEST GPU as compared to NEST. This is due to the fact that NEST GPU builds the network in the RAM and thereafter copies the constructed model to GPU memory. This additional step represents the bottleneck of the network construction phase using NEST GPU. However, since the build time is independent of the biological simulation time, it can be regarded as an overhead with decreasing relevance for longer biological times. A future integration of the network construction phase into the GPU memory could strongly decrease this contribution.

In terms of simulation time, NEST GPU shows a remarkable performance (Figure 8A). Simulations of the multi-area model in its ground state achieved a simulation time of 6.5 s per second of biological activity, reaching a speed-up factor of 2.4 compared to NEST. In the metastable state, NEST GPU reached 15.3 s of simulation time per second of biological activity which is approximately 3.1× faster than NEST simulations. Future work can further improve upon this performance: firstly, if each node of the HPC cluster were equipped with more than one GPU, the communication time, and with it the simulation time, would diminish. Secondly, the same simulation performed with a more recent GPU hardware (e.g., NVIDIA A100 GPUs) would permit not only faster simulations but also the possibility to simulate more than one area of the model on the same GPU thanks to enhancements of the GPU memory.

From the Results section it can be observed that the most relevant differences in the performance of NEST and NEST GPU in the simulation of the multi-area model are related to the contribution of the spike communication to the total simulation time. NEST uses a round-robin distribution of the nodes among MPI processes, and a two-tier connection infrastructure for communicating spikes. This infrastructure differentiates between data structures on the presynaptic side, i.e., the MPI process of the sending neuron, and the postsynaptic side, i.e., the MPI process of the receiving neuron. By using the blocking MPI Alltoall, spikes, which are stored in MPI buffers, are routed across MPI processes from pre- to postsynaptic neurons. In the implementation described by?, a spike having target neurons on different threads necessitated communication of spike copies to all these threads. Furthermore, this implementation only allowed MPI buffers to grow, but not to shrink. Albers et al. (2022) identified that this puts unnecessary strain on the MPI communication. They therefore introduced spike compression which only sends one spike to each target MPI process, which has the necessary knowledge on the target threads saved in an additional data structure. The problem of buffer size is solved *via* introducing the possibility of dynamically shrinking and growing the MPI buffers.

Kumar et al. (2010) and Hines et al. (2011) propose and compare several strategies for spike-exchange on systems including up to 128 K communication end-points (fine-grained BlueGene/P cores) leveraging a communication infrastructure based on non-blocking neighborhood collectives. The proposed approach has several points of strength that have not yet been exploited in this paper, for several reasons. First, communication steps are performed every ms (the minimum axo-synaptic delay in their model), while the integration step is set at 0.1 ms. Some of the authors of the present paper already exploited this strategy (e.g., in Pastorelli et al., 2019) demonstrating its substantial merit in reducing the communication/computation time ratio. However, the minimal connection delay in the 32-area model under consideration is not higher than the integration step, so this prevents the application of the method in the current paper. However, this will be considered for multi-area models with inter-areal connection delays substantially longer than the integration step. Second, in Kumar et al. (2010) and Hines et al. (2011) communication and computation are overlapped by further dividing the communication step in two alternating temporal steps (A and B, with spikes produced during the time window A sent during the B window, and vice versa). Substantial minimal inter-areal connection delays are again a precondition for the overlapping of computation and communication, but it must also be supported by adequate infrastructure in the simulation engine. This technique further reduces the overhead of communication down to values that are comparable with the computation cost even for highly simplified neural integration models. The technique should be surely considered for implementation in NEST GPU and NEST.

Concerning the merit of distributing the neurons among nodes according to their spatial locality (as in the NEST GPU implementation), there are several substantial differences between the spike-exchange algorithmic exploration proposed by Hines et al. (2011) (from 8 to 128 K small memory footprint MPI end-points in the BlueGene/P system) and our discussion that uses as end-points of MPI communication 32 large memory node systems. Hines et al. (2011) analyze the effect of round-robin vs. a consecutive distribution of neurons among processing nodes for models with random vs. local connectivity, in two spiking rate regimes, named “Noburst” and “Burst.” In the first one each neuron of the network fires with a uniform distribution over the entire simulation time interval, whereas in the second regime groups of contiguous neurons successively fire at five times their normal rate for a 50 ms period. On a system with 16 K communication end-points, they demonstrated that the consecutive distribution is highly advantageous for locally connected networks with homogeneous firing rates, while it is only moderately advantageous when all neurons on a processor show the bursting regime. In our case, with larger memory per node and in general for horizontal projections strongly decaying with spatial distance, mapping the laterally incoming synapses on the memory of a single GPU eliminates the need to use collective communications for a much larger fraction of spikes than when mapping a structured network on a system with 16K communication end-points. Indeed, the average ratio between the number of spikes that an area sends to all the other areas and the total number of spikes that it emits is around 3%, with a maximum across areas of around 16% (see Supplementary Material).

Regarding NEST GPU performance on a learning case (i.e., on a network model that employs plastic synapses), in Golosio et al. (2021b) we evaluated the library's performance in the simulation of networks with spike-timing-dependent plasticity (STDP) (Gütig et al., 2003) on a single GPU. In general, multi-GPU/MPI simulation performance can significantly depend on the way synaptic parameters of STDP connections between neurons on different MPI processes are updated. The availability of presynaptic spikes and synaptic representation on the same process as the target neurons, as in NEST GPU (and NEST), enables efficient weight updates because they can be managed locally. However, simulation of plastic networks will be covered in future work.

The inclusion of NEST GPU into the NEST Initiative facilitates further integration with the NEST simulator, opening it up to GPU-based spiking neural network simulations. Currently there is ongoing work oriented to an adaptation of the models to be consistent with the NEST simulator, and a software interface has also been developed (Golosio et al., 2020) which enables creating NEST-NEST GPU hybrid networks. Indeed, as reported in Golosio et al. (2021b), the Python interfaces of NEST and NEST GPU are highly similar, making the porting of NEST scripts to the new simulator quite simple. Not only the possibility of using GPU hardware, but also the optimized MPI algorithm for spike communication will greatly improve user experience in simulating large-scale spiking neural networks. In fact, as shown in Figure 8, the time reduction in the communication between MPI processes is the main contributor to the better performance of NEST GPU compared to NEST. A speed-up in the neuron updates and delivery of local spikes is also present, and can be further enhanced with the use of more performant GPU-based HPC solutions.

In summary, the NEST GPU simulator (Golosio et al., 2021b) is able to outperform NEST in the state propagation phase of the simulation of a large-scale spiking model, and this speed-up can be essential for simulations covering long stretches of biological time. The performance might be even further enhanced with the help of the latest GPU hardware, which could lead to a steeper performance difference between CPU-based simulators and GPU-based ones. Indeed the use of multi-GPU nodes in a cluster, together with the increase in GPU memory and therefore the possibility of allocating more neurons on a single GPU card, would allow a considerable reduction in the spike communication time. More generally, the GPU industry is growing rapidly, with excellent prospects for the performance of future cards, which from generation to generation significantly increase performance.

## Data Availability Statement

The datasets presented in this study can be found in online repositories. The names of the repository/repositories and accession number(s) can be found below: https://github.com/gmtiddia/ngpu_multi_area_model_simulation.

## Author Contributions

GT, JA, and JP performed the simulations and data analysis with guidance by JS, BG, and SvA. JP, JA, JS, and SvA contributed to the development of the NEST implementation of the multi-area model. BG, FS, GT, EP, VF, and PP contributed to the development of the NEST GPU implementation of the multi-area model. GT, BG, and SvA wrote the first manuscript draft. JS, GT, JA, EP, PP, VF, and SvA revised the manuscript. BG and SvA supervised the project. All authors have read and approved the final manuscript.

## Funding

This study was supported by the European Union's Horizon 2020 Framework Programme for Research and Innovation under Specific Grant Agreements No. 945539 (Human Brain Project SGA3) and No. 785907 (Human Brain Project SGA2), the Priority Program 2041 (SPP 2041) Computational Connectomics of the German Research Foundation (DFG), the Helmholtz Association Initiative and Networking Fund under project number SO-092 (Advanced Computing Architectures, ACA), the Joint Lab Supercomputing and Modeling for the Human Brain, and the INFN APE Parallel/Distributed Computing laboratory. We acknowledge the use of Fenix Infrastructure resources, which are partially funded from the European Union's Horizon 2020 research and innovation programme through the ICEI project under the Grant Agreement No. 800858. Open access publication funded by the Deutsche Forschungsgemeinschaft (DFG, German Research Foundation) – 491111487.

## Conflict of Interest

The authors declare that the research was conducted in the absence of any commercial or financial relationships that could be construed as a potential conflict of interest.

## Publisher's Note

All claims expressed in this article are solely those of the authors and do not necessarily represent those of their affiliated organizations, or those of the publisher, the editors and the reviewers. Any product that may be evaluated in this article, or claim that may be made by its manufacturer, is not guaranteed or endorsed by the publisher.

## Footnotes

^1^The distributions are available at https://github.com/gmtiddia/ngpu_multi_area_model_simulation/tree/main/analysis/dist_plots/Areas.
