# Supplementary material for: Fast Simulation of a Multi-Area Spiking Network Model of Macaque Cortex on an MPI-GPU Cluster
Source: Front Neuroinform. 2022 Jul 4;16:883333. doi: 10.3389/fninf.2022.883333 (PMC9289599; doi:10.3389/fninf.2022.883333)
Supplement: Supplementary file 1 [file Data_Sheet_1.PDF]

Supplementary Material

1 EARTH MOVER'S DISTANCE

The Earth Mover's Distance (EMD) is a metric that evaluates the distance between two probability distributions. It is equivalent to the 1st Wasserstein distance between two distributions.

The 1st Wasserstein distance is defined as follows (Panaretos and Zemel, 2019; Frohmader and Volkmer, 2021): let χ be a metric space endowed with a metric d and μ and ν be two probability measures on χ. Also let $\Gamma(\mu, \nu)$ be the set of probability measures γ on $\chi \times \chi$ so that the measure γ has marginals μ and ν on each axis. The pth-Wasserstein distance can be written as

$$W_p(\mu, \nu) = \left(\inf_{\gamma \in \Gamma(\mu,\nu)} \int_{\chi \times \chi} d(x, y)^p d\gamma(x, y) \right)^{1/p} \tag{S1}$$

This relation yields the given interpretation of the Earth Mover's Distance. In particular, given a $\gamma \in \Gamma(\mu, \nu)$ and two locations (x, y), the quantity $d(x, y)^p$ quantifies the amount of work necessary to move a unit of mass from x to y, and the infimum of the integral thus returns the minimum amount of work needed to reshape μ distribution into ν.

Furthermore, equation (S1) can be rewritten when $\chi = \mathbb{R}^1$ (ergo $d(x, y) = |x - y|$). Letting F_X be the cumulative distribution function (CDF) of a distribution X and $F_X^{-1}(q) = \inf\{x : F_X(x) \geq q\}, q \in (0, 1)$ the quantile distribution of X (i.e. the inverse cumulative distribution), it is shown in Ramdas et al. (2017) that

$$W_p(\mu, \nu) = \left(\int_0^1 |F_\mu^{-1}(\alpha) - F_\nu^{-1}(\alpha)|^p d\alpha \right)^{1/p}. \tag{S2}$$

In the particular case of the 1st Wasserstein distance (i.e. $p = 1$), as discussed in Vallender (1974), equation (S2) reduces to

$$W_1(\mu, \nu) = \text{EMD}(\mu, \nu) = \int_{\mathbb{R}} |F_\mu(t) - F_\nu(t)| dt. \tag{S3}$$

that is the equation used by the Python scientific library SciPy (Virtanen et al., 2020) (version 1.5.2) to compute this metric with the `scipy.stats.wasserstein_distance` function, which takes the values of the distributions as input, computes their CDF and finally returns the result of the integral (S3).

2 EMD BOX PLOTS

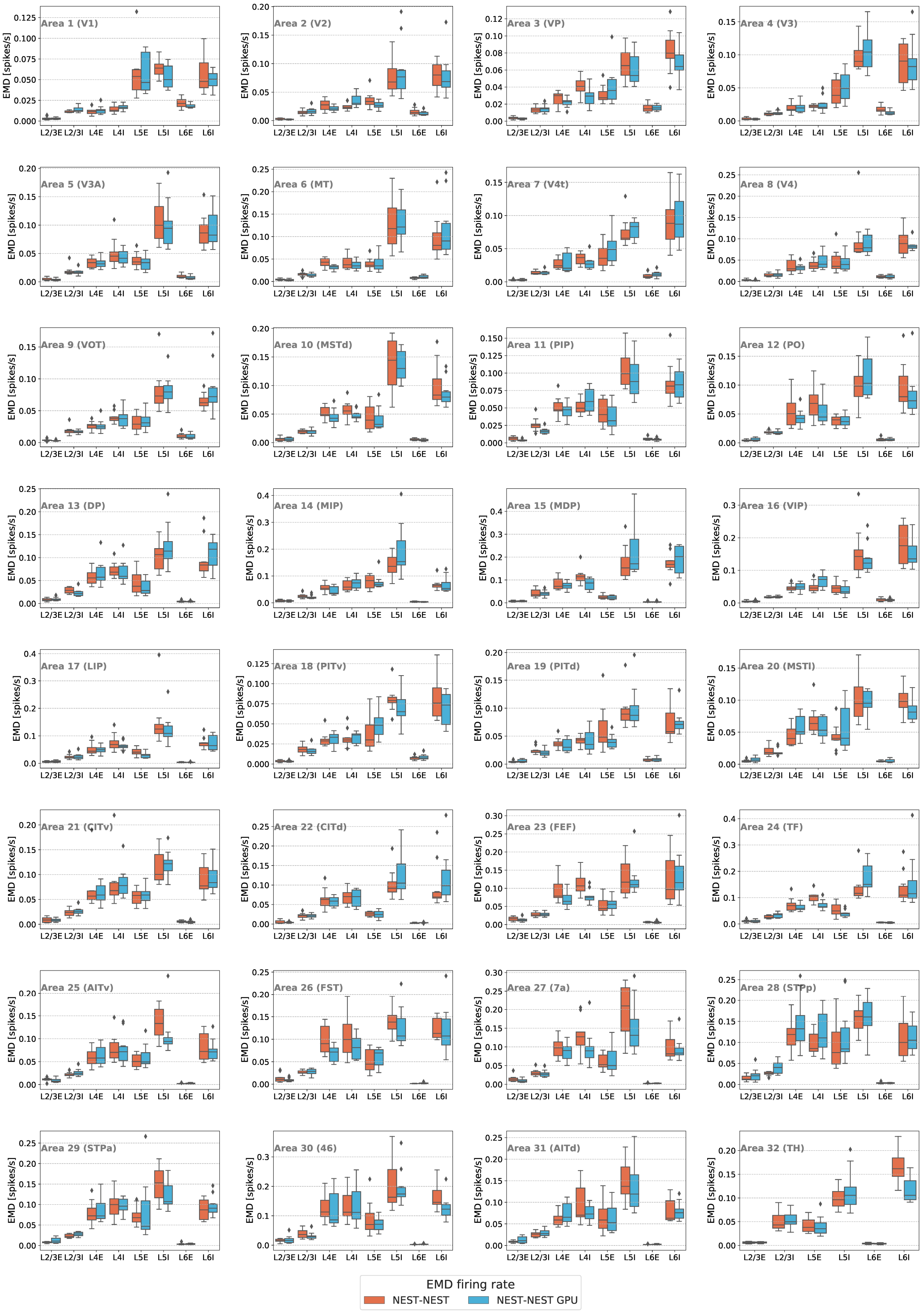

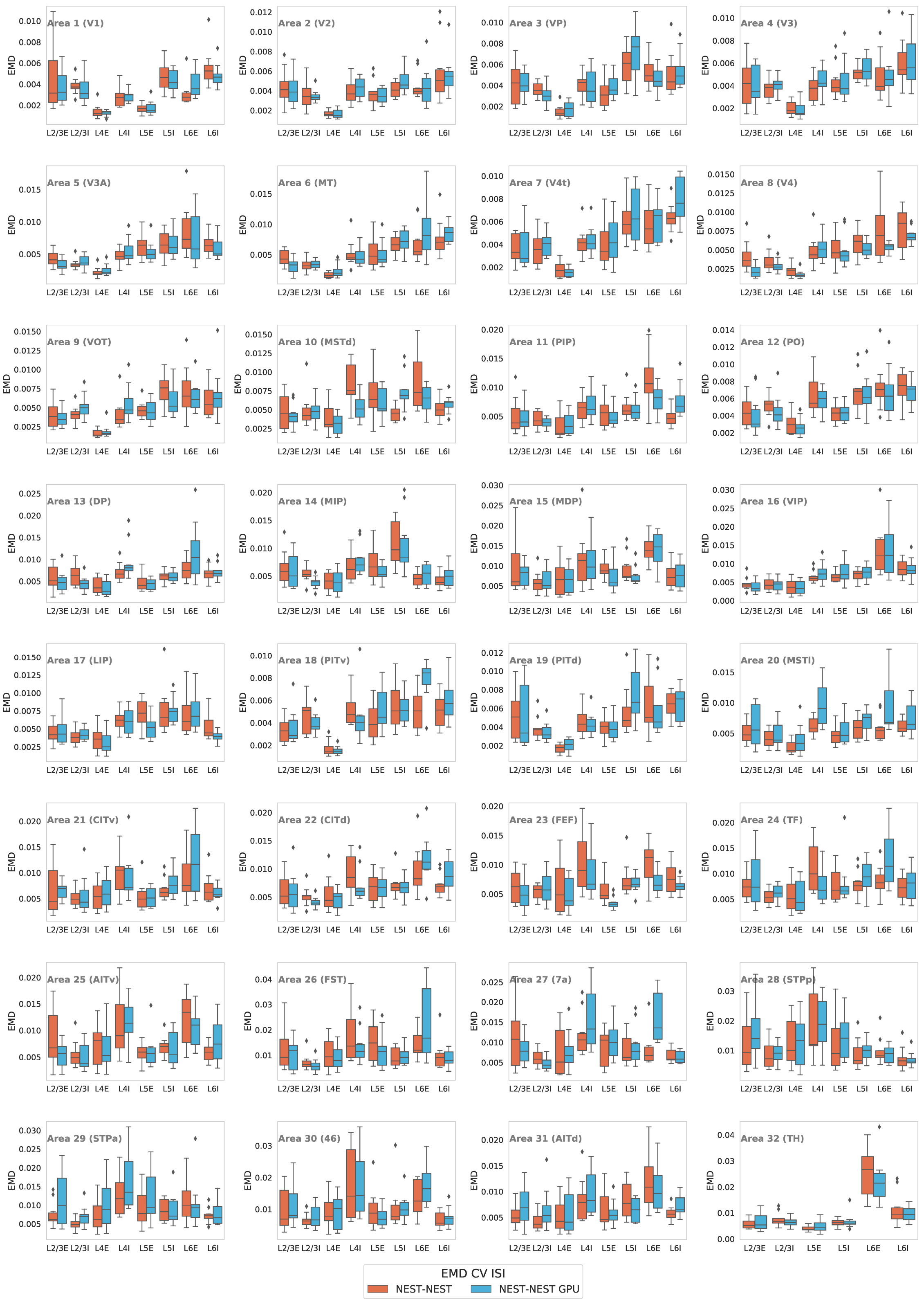
Area 1 (V1)
Area 2 (V2)
Area 3 (VP)
Area 4 (V3)
Area 5 (V3A)
Area 6 (MT)
Area 7 (V4t)
Area 8 (V4)
Area 9 (VOT)
Area 10 (MSTd)
Area 11 (PIP)
Area 12 (PO)
Area 13 (DP)
Area 14 (MIP)
Area 15 (MDP)
Area 16 (VIP)
Area 17 (LIP)
Area 18 (PITv)
Area 19 (PITd)
Area 20 (MSTl)
Area 21 (CITv)
Area 22 (CITd)
Area 23 (FEF)
Area 24 (TF)
Area 25 (AITv)
Area 26 (FST)
Area 27 (7a)
Area 28 (STPp)
Area 29 (STPa)
Area 30 (46)
Area 31 (AITd)
Area 32 (TH)
EMD
L2/3E L2/3I L4E L4I L5E L5I L6E L6I
EMD CV ISI
NEST-NEST
NEST-NEST GPU

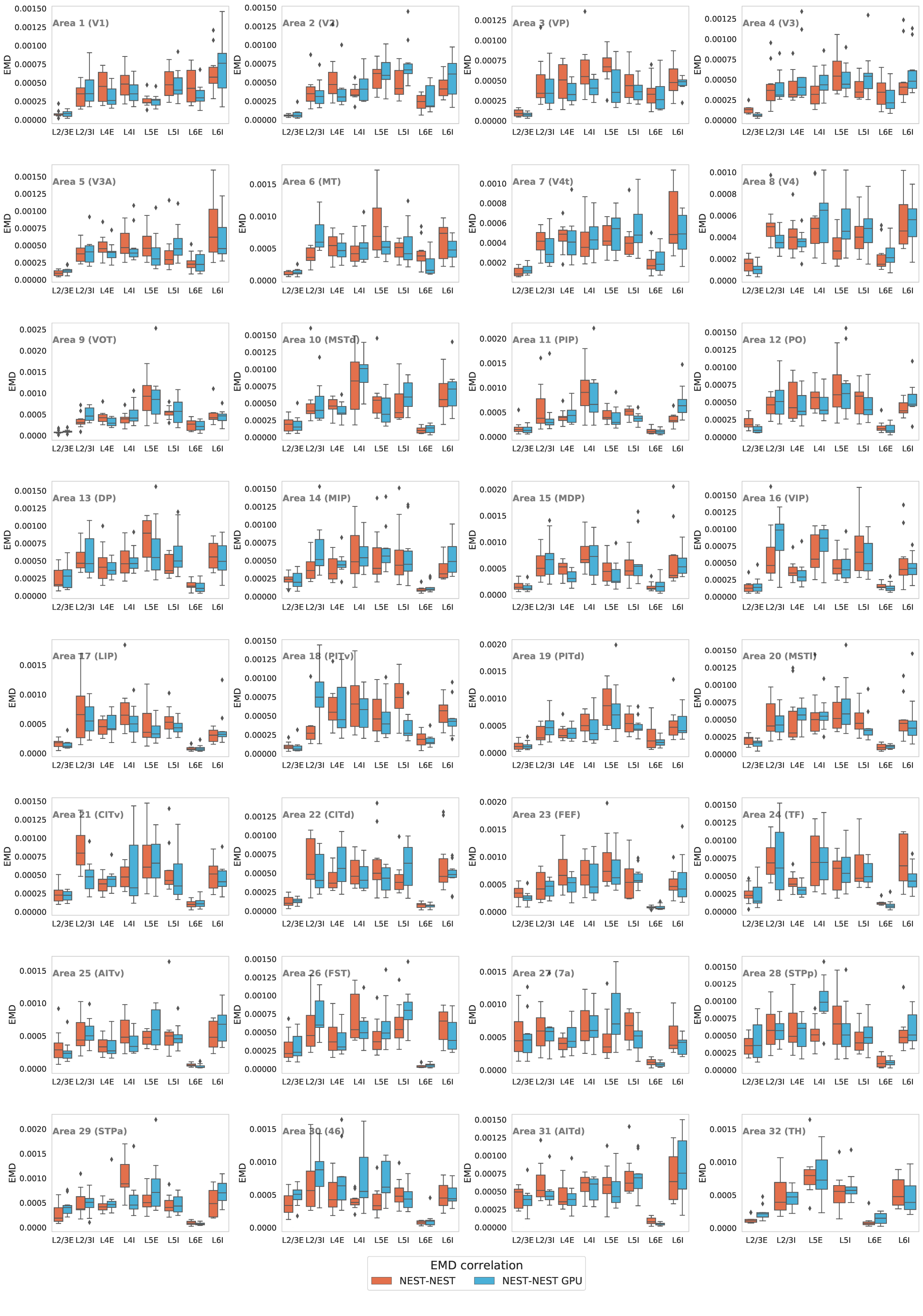

Figure S1. Earth Mover's Distance between distributions of firing rate, CV ISI and correlation of the spike trains obtained from all the areas of the model in the ground state simulated with NEST and NEST GPU.

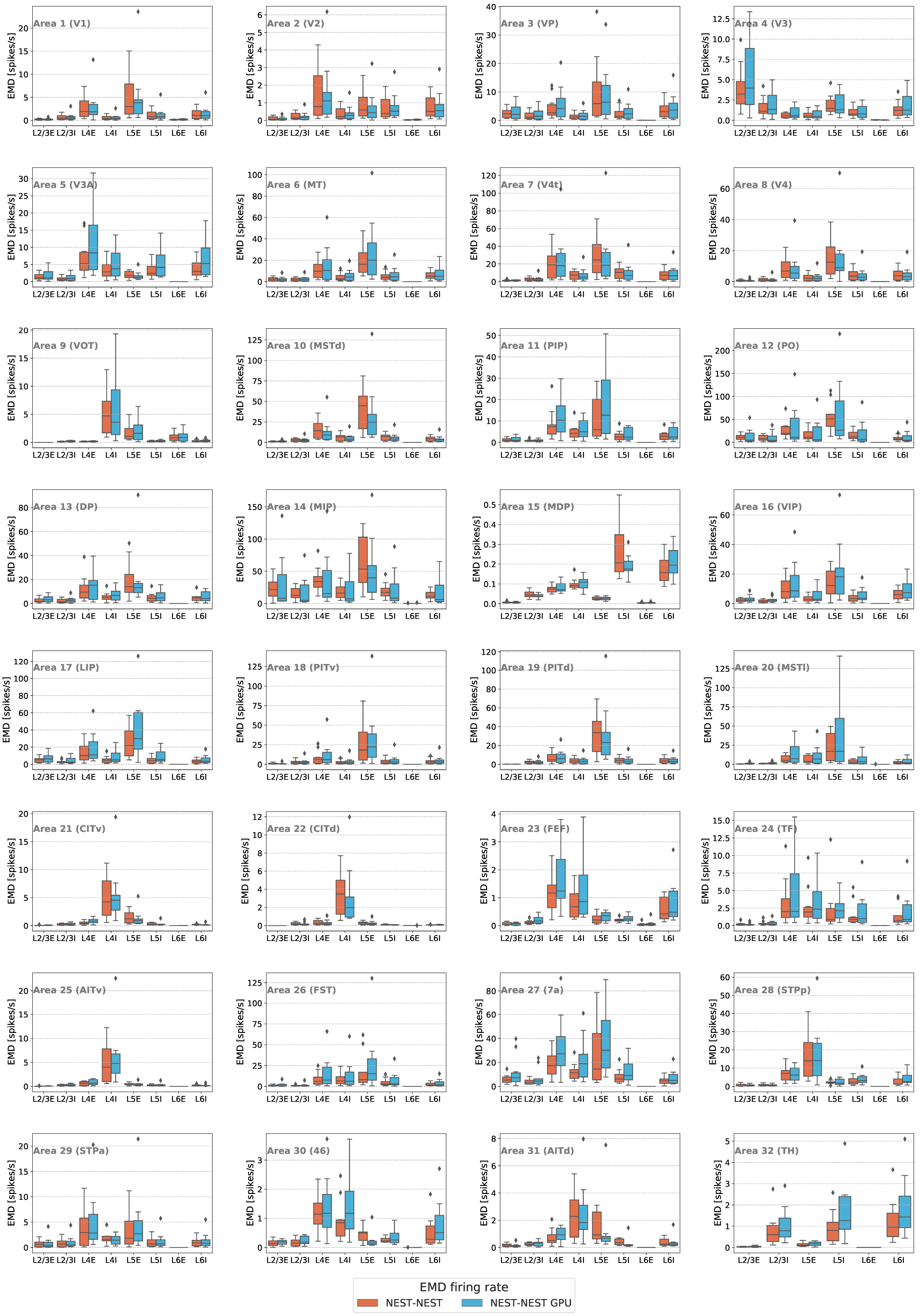
Area 1 (V1)
Area 2 (V2)
Area 3 (VP)
Area 4 (V3)
Area 5 (V3A)
Area 6 (MT)
Area 7 (V4t)
Area 8 (V4)
Area 9 (VOT)
Area 10 (MSTd)
Area 11 (PIP)
Area 12 (PO)
Area 13 (DP)
Area 14 (MIP)
Area 15 (MDP)
Area 16 (VIP)
Area 17 (LIP)
Area 18 (PITv)
Area 19 (PITd)
Area 20 (MSTl)
Area 21 (CITv)
Area 22 (CITd)
Area 23 (FEF)
Area 24 (TF)
Area 25 (AITv)
Area 26 (FST)
Area 27 (7a)
Area 28 (STPp)
Area 29 (STPa)
Area 30 (46)
Area 31 (AITd)
Area 32 (TH)
EMD [spikes/s]
L2/3E L2/3I L4E L4I L5E L5I L6E L6I
EMD firing rate
NEST-NEST
NEST-NEST GPU

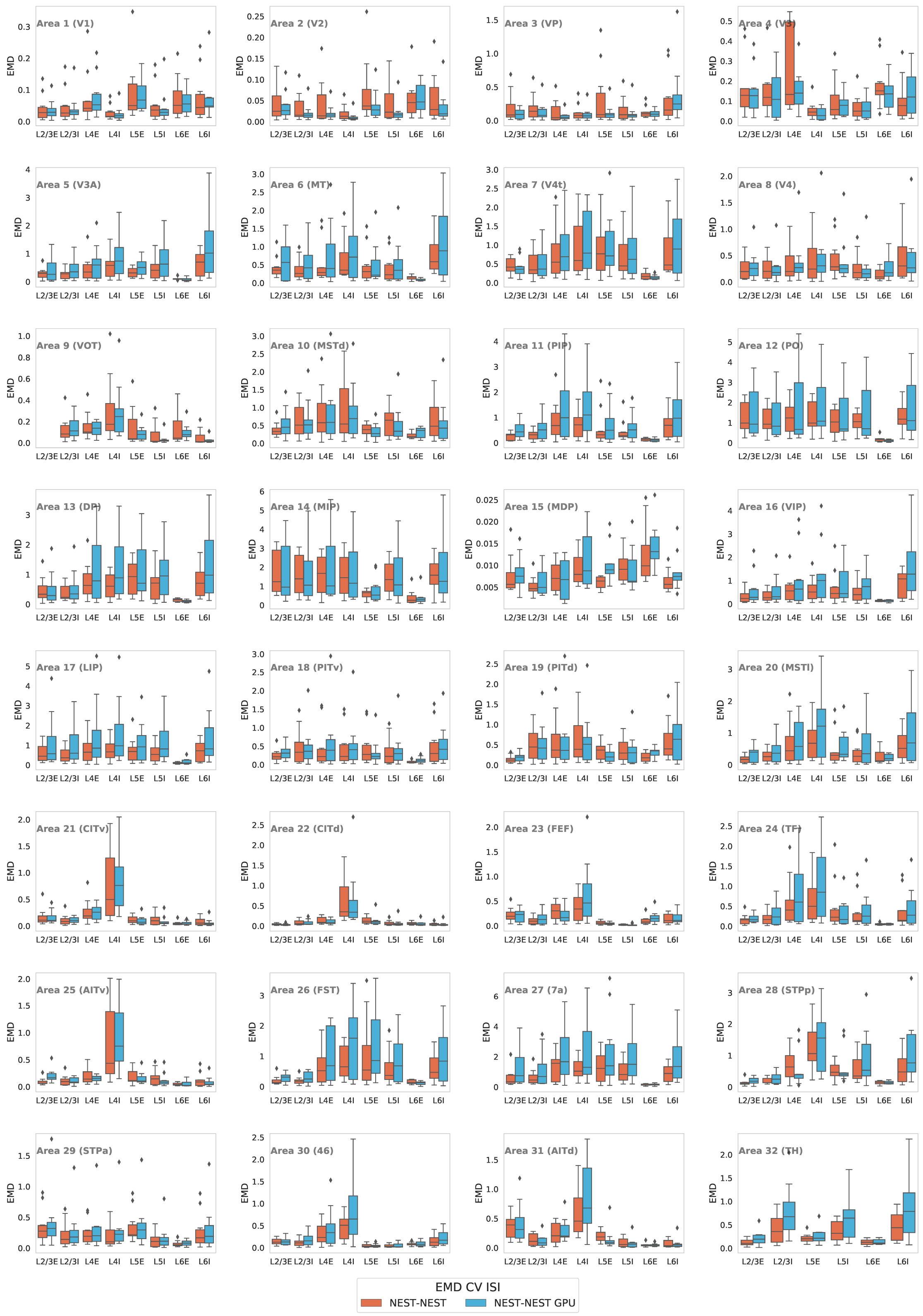
Area 1 (V1)
Area 2 (V2)
Area 3 (VP)
Area 4 (V3)
Area 5 (V3A)
Area 6 (MT)
Area 7 (V4t)
Area 8 (V4)
Area 9 (VOT)
Area 10 (MSTd)
Area 11 (PIP)
Area 12 (PO)
Area 13 (DP)
Area 14 (MIP)
Area 15 (MDP)
Area 16 (VIP)
Area 17 (LIP)
Area 18 (PITv)
Area 19 (PITd)
Area 20 (MSTl)
Area 21 (CITv)
Area 22 (CITd)
Area 23 (FEF)
Area 24 (TF)
Area 25 (AITv)
Area 26 (FST)
Area 27 (7a)
Area 28 (STPp)
Area 29 (STPa)
Area 30 (46)
Area 31 (AITd)
Area 32 (TH)
EMD
L2/3E L2/3I L4E L4I L5E L5I L6E L6I
EMD CV ISI
NEST-NEST
NEST-NEST GPU

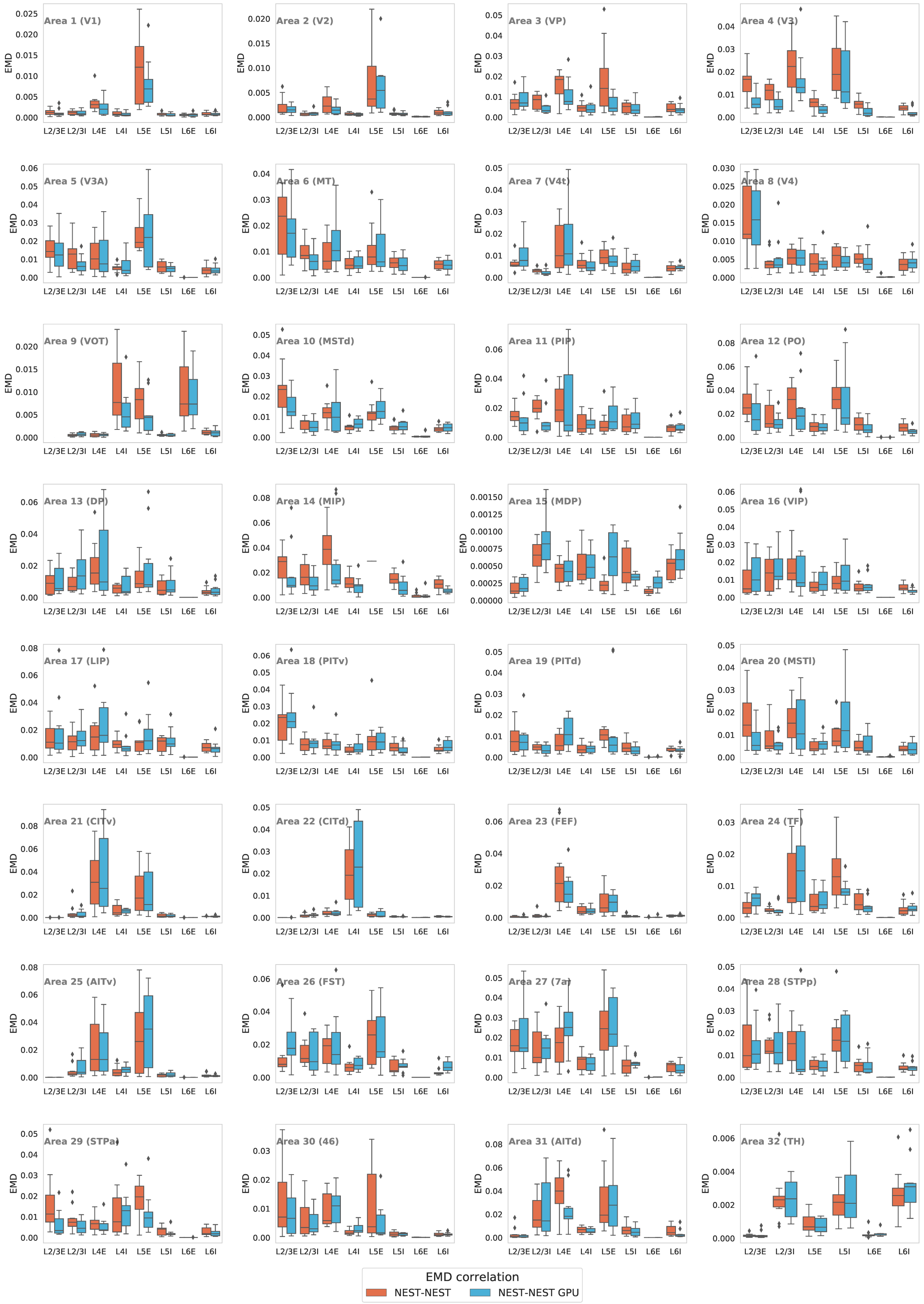

Figure S2. Earth Mover's Distance between distributions of firing rate, CV ISI and correlation of the spike trains obtained from all the areas of the model in the metastable state simulated with NEST and NEST GPU.

3 SPIKES DELIVERED ACROSS MODEL AREAS

In this section are shown some additional information about the spikes delivered within the areas of the model (ergo between different MPI processes). Indeed, the simulations employ 32 nodes with one MPI process each, in every one of which is simulated one area of the multi-area model. Figure S3 shows that the vast majority of the spikes emitted in a second of biological time are delivered within the same area, and only a small fraction of the spikes is delivered to different areas because the inter-area connections represent only a minority of the total connectivity of the model (Schmidt et al., 2018). Therefore, the NEST GPU neuron distribution, which exploits spatial locality, strongly reduce the amount of spikes that have to be carried out by communication between MPI processesand thus contribute to the overall simulation time reduction.

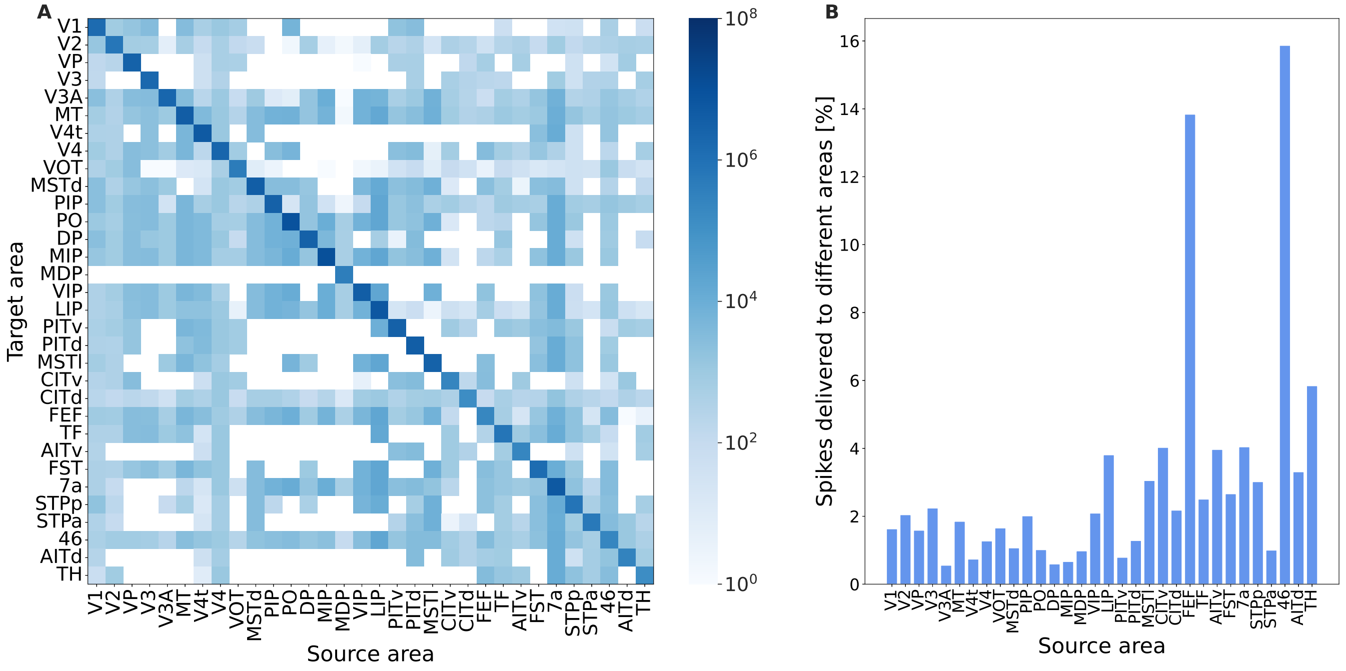

Figure S3. Spikes delivered in a simulation of the full-scale multi-area model in the metastable state. **(A)** Heatmap of the number of spikes delivered from each area of the model for a second of biological time. The diagonal elements show the total number of spikes fired by the neurons within the model area, whereas the off-diagonal elements show the number of spikes delivered to different areas of the model. **(B)** Fraction of spikes delivered to different areas of the model (i.e. to different MPI processes), obtained with the ratio between the number of spikes sent by a source area to every other area of the model and the total number of spikes emitted by the source area neurons.

REFERENCES

Frohmader, A. and Volkmer, H. (2021). 1-Wasserstein distance on the standard simplex. *Algebraic Statistics* 12, 43–56. doi:10.2140/astat.2021.12.43

Panaretos, V. M. and Zemel, Y. (2019). Statistical aspects of Wasserstein distances. *Annual Review of Statistics and its Application* 6, 405–431. doi:10.1146/annurev-statistics-030718-104938

Ramdas, A., Trillos, N. G., and Cuturi, M. (2017). On Wasserstein two-sample testing and related families of nonparametric tests. *Entropy* 19. doi:10.3390/e19020047

Schmidt, M., Bakker, R., Hilgetag, C. C., Diesmann, M., and van Albada, S. J. (2018). Multi-scale account of the network structure of macaque visual cortex. *Brain Structure and Function* 223, 1409–1435. doi:10.1007/s00429-017-1554-4

Vallender, S. S. (1974). Calculation of the Wasserstein distance between probability distributions on the line. *Theory of Probability & Its Applications* 18, 784–786. doi:10.1137/1118101

Virtanen, P., Gommers, R., Oliphant, T. E., Haberland, M., Reddy, T., Cournapeau, D., et al. (2020). SciPy 1.0: Fundamental Algorithms for Scientific Computing in Python. *Nature Methods* 17, 261–272. doi:10.1038/s41592-019-0686-2
